# Prevalence of violence to others among individuals with schizophrenia in China: A systematic review and meta-analysis

**DOI:** 10.3389/fpsyt.2022.939329

**Published:** 2022-07-22

**Authors:** Yi Guo, Xianmei Yang, Dan Wang, Ruoxin Fan, Yiying Liang, Rongke Wang, Hu Xiang, Yuanyuan Liu, Xiang Liu

**Affiliations:** ^1^Department of Epidemiology and Biostatistics, West China School of Public Health and West China Fourth Hospital, Sichuan University, Chengdu, China; ^2^Sichuan Mental Health Center, The Third Hospital of Mianyang, Mianyang, China; ^3^Department of Health Behavior and Social Medicine, West China School of Public Health and West China Fourth Hospital, Sichuan University, Chengdu, China

**Keywords:** meta-analysis, prevalence, schizophreina, spatial distribution, violence

## Abstract

**Background:**

Violence to others (hereinafter referred to as “violence-TO”) is common in individuals with schizophrenia. The reported prevalence of violence-TO among schizophrenics ranges widely in existing studies. Improved prevalence estimates and identification of moderators are needed to guide future management and research.

**Methods:**

We searched EBSCO, EMBASE, Medline, PubMed, Science Direct, Web of Science, CNKI, VIP, WANFANG data, and CBM for relevant articles published before June 5, 2022. Meanwhile, violence-TO was summarized into four categories: (a) violence-TO on the reviews of official criminal or psychiatric records (type I); (b) less serious forms of violence-TO (type II); (c) physical acts causing demonstrable harm to victims (type III); (d) homicide (type IV). We did meta-analysis for the above types of violence-TO, respectively, and applied subgroup analyses and meta-regression analyses to investigate the source of heterogeneity.

**Results:**

A total of 56 studies were eligible in this study and 34 of them were high-quality. The prevalence of type I to type IV in individuals with schizophrenia in China was 23.83% (95% CI: 18.38–29.75%), 23.16% (95% CI: 8.04–42.97%), 17.19% (95%CI: 8.52–28.04%), and 0.62% (95% CI: 0.08–1.54%) respectively. The results of the subgroup analysis showed that the prevalence of type I was higher among subjects in the inland than in the coastal non-economic zone, while the prevalence of type III was the highest in the coastal economic zone, followed by the inland region and the lowest in the coastal non-economic zone. The results of multivariate meta-regression analyses showed that: patient source in type I (β = 0.15, *P* < 0.01), patient source (β = 0.47, *P* < 0.01), and proportion of male (β = 0.19, *P* < 0.01) in type II, age (β = 0.25, *P* < 0.01), and GDP per capita (β = 0.05, *P* = 0.01) in type III were statistically significant.

**Conclusion:**

The prevalence of different types of violence-TO and their influencing factors varied. Therefore, the authorities should take different management measures. In addition to individual factors, regional factors may also affect violence-TO, which suggests the need for a multi-sectorial approach to prevention and treatment for subjects in different regions and adopting targeted control strategies.

**Systematic Review Registration:**

[www.ClinicalTrials.gov], identifier [CRD42021269767].

## Background

According to the Global Burden of Disease Study, as of 2019, there were approximately 5.5 million individuals with schizophrenia in China, ranking first in the world (1). Schizophrenia is the most serious mental disorder, which can be associated with aggressive and violent behavior. Several large cohort studies indicated an increase in violence in schizophrenics (2, 3). Violence in individuals with schizophrenia can be explained by demographic and personality factors, psychopathological symptoms, genetic or biological markers, and neuroimages (4–7). Despite great efforts have been made to study on markers associated with violence and schizophrenia, there haven’t been consistent findings so far to explain the association (5). Violence experienced by subjects with mental disorders included three forms: violence to others (hereinafter referred to as “violence-TO”), violent self-victimization, and violent victimization by others (8). Among them, violence-TO, which has been focused on by enormous studies in the past few decades, brings in a series of adverse effects, such as harming others, discrimination and stigmatization, etc. (9).

Exploring the prevalence of violence-TO in individuals with schizophrenia is vital for more effective prevention and health resource allocation, but at present, there are still the following problems remaining in the current studies: First, there is a lack of a unified definition and measurement of violence (9). The definitions of violence varied from research to research. For example, some included threats and verbal assault (9), while others only considered physical violence (10), or collectively called self-victimization and violence-TO “violence” (3). Meanwhile, violence was measured by various kinds of instruments, like Broset-Violence-Checklist (BVC), Modified Overt Aggression Scale (MOAS), Historical, Clinical, Risk management (HCR-20), and so on. Consequently, different studies focused on different violence. Second, schizophrenia is a heterogeneous clinical syndrome and individuals with this disorder can be extremely different from variables related to violence-TO action (4). Therefore, violence-TO *per se* is also heterogeneous in origin, which makes it challenging to deal with both in research and in clinical practice (11). Third, for the original studies, the reported prevalence varies widely for study aims, designs, and methods. As a result, it is hard to draw a general conclusion, to explore the prevalence of violence-TO in individuals with schizophrenia (12).

Probably influenced by the above reasons, only three systematic reviews have summarized the prevalence of violence in individuals with schizophrenia so far (13–15). These studies still have certain limitations: first, they focused on violence in general terms, which didn’t distinguish between violence-TO and self-victimization, and did not include a comprehensive range of types of violence-TO; second, most of these studies focused on inpatients, which limited the inference of their conclusions; finally, these studies were highly heterogeneous, but they didn’t deepen into the heterogeneity source.

In view of the above limitations, this study proposed to systematically estimate the prevalence of violence-TO among schizophrenics in China, and to explore the sources of heterogeneity. First, compared with previous studies, the violence-TO types included in our study were more comprehensive. According to the studies of Douglas et al. (9), violence-TO in this study was summarized into four categories: (a) violence-TO on the basis of reviews of official criminal or psychiatric records (hereinafter referred to as “”type I”); (b) less serious forms of violence-TO, such as minor physical acts (i.e., pushing) or verbal behavior (i.e., threats to harm someone) (hereinafter referred to as “”type II””); (c) physical acts that caused demonstrable harm to victims (hereinafter referred to as “type III”); (d) homicide (hereinafter referred to as “type IV”). Second, this study focused on both inpatients and non-inpatients. Finally, in addition to individual-level variables reported in the original articles, our study also included regional indicators for the subgroup analysis and the meta-regression analysis, and deeply look into the sources of heterogeneity as well as the possible influencing factors of various types of violence-TO.

To enrich the research on the prevalence of violence-TO among schizophrenics in China in a new viewpoint, this study remedies the limitations of previous studies that reported generally or non-heterogeneously on the prevalence of violence-TO, only focused on inpatients alone, and lacked the exploration to heterogeneous sources and influencing factors of violence-TO. We aimed at determining better estimates of the prevalence of violence-TO in individuals with schizophrenia in China, by including comprehensive range of violent behavior and patients from different sources for better representativeness. Meanwhile, we target at identifying the extent to which moderators account for heterogeneity by considering both regional and individual level factors, and exploring the geographic characteristics of violence-TO by subgroup and spatial analysis, thereby providing a reference for the management and prevention of violence-TO in the schizophrenia population.

## Materials and methods

### Study registration

The aim of the meta-analysis was to provide a broad overview of prevalence of violence-TO among individuals with schizophrenia in China. It was undertaken according to PRISMA criteria (16). Please see the checklist in Supplementary Table 1. This study had been registered in PROSPERO with number CRD42021269767.

### Search strategy

We searched EBSCO, EMBASE, Medline, PubMed, Science Direct, Web of Science, CNKI, VIP, WANFANG data, and CBM, without the language restriction, using the terms (“violence” OR “risk behavior” OR “illegal behavior” OR “aggression” OR “criminal behavior” OR “Injury” or “offensive behavior” OR “homicidal behavior” OR “agitation”) AND (“schizophrenia”) AND (“China” OR “Chinese”), along with manual retrieval, in order to collect literatures related to violence-TO among schizophrenics in China comprehensively. The time frame of the literature search was published until June 5, 2022. Please see the search strategy in Supplementary Table 2.

### Inclusion and exclusion criteria

All potentially eligible studies were examined. The following criteria were established to select relevant articles.

Inclusion criteria: (a) cross-sectional or cohort studies (only the baseline data were included); (b) individuals in China; (c) a diagnosis of schizophrenia according to International Classification of Diseases, 9th Edition, 10th Edition or 11th Edition (ICD-9, ICD-10 or ICD-11), Classification and Diagnostic Criteria of Mental Disorders in China, 1st Edition, 2nd Edition or 3rd Edition (CCMD-1, CCMD-2 or CCMD-3) or Diagnostic and Statistical Manual of Mental Diseases, 2nd Edition, 3rd Edition, 4th Edition or 5th Edition (DSM-II, DSM-III, DSM-IV, DSM-V); (d) a clear definition or description of violence-TO in the original studies, and the violence-TO could be classified into the 4 categories described above according to the definition in the original studies; (e) reporting data on the prevalence of violence-TO or providing data available to calculate the prevalence; (f) for multiple articles that used data from the same investigation, the one with the most comprehensive results was kept; (g) violence-TO should occur after the diagnosis of schizophrenia.

Exclusion criteria: (a) reviews or abstracts; (b) trials; (c) studies that did not differentiate violence-TO from violent self-victimization; (d) unusable information; (e) researches with unclear study sites; (f) researches with low quality.

Two researchers (YG and XY) individually screened titles and abstracts, and then read full texts of relevant publications for eligibility. Any disagreement in literature selection was resolved by consulting the senior investigator (DW).

### Quality assessment

We used Newcastle Ottawa Scale (NOS) to assess the quality of studies. There were 6 items for cross-sectional study with a full score of 7, and 8 items for cohort study with a full score of 9. Studies were divided into 3 groups according to the following criteria: (a) score < 4; (b) score ≥ 4 and score < 7; (c) score ≥ 7. Researches in group a were taken for low quality and were excluded from the study. And those in group b and group c were considered to be eligible studies and therefore were included. Those in group c were deemed high-quality (17). Quality assessment was conducted independently by RF and YL and any disagreement was resolved by consulting the senior investigator (RW).

### Data extraction

Two researchers (RF and YLia) independently extracted the following information using a pre-made data collection sheet: first, the internal data included in the original studies, such as study information, participant characteristics, prevalence of violence-TO, etc.; second, regional-level indicators potentially associated with violence-TO from *China Statistical Yearbook* and *China Health Statistics Yearbook* according to the publication year (research time was replaced by the publication year due to unavailable information) and the study sites, such as GDP per capita, unemployment rate, total burden coefficient, illiteracy rate, beds per 1,000 population in health institutions.

### Data analysis and risk of bias

Meta-analysis was conducted using the meta and metafor packages of R Project Version 4.0.3, and we calculated pooled estimates of prevalence using Freeman-Turkey-double-arcsine transformation to correct for non-normally distributed raw data (18). Considering various designs across studies, DerSimonian-Laird random effect model was utilized in all analyses. Heterogeneity was tested by Cochran’s *Q*-test and *I*^2^ statistics (a value of 0% indicates no observed heterogeneity, and larger values show increasing heterogeneity. It is usually assigned low, moderate and high to *I*^2^-values of 25%, 50%, 75%) (19, 20). Publication bias was tested by the funnel plot and Egger linear regression test for analyses with more than 10 studies included. “Trim and Fill” method was used to adjust for publication bias (21). The pooled estimates of prevalence in each province were calculated, and a statistical map was drawn through ggplot2 package for visualization of spatial distribution.

Subgroup analysis was used to compare the differences in the violence-TO prevalence in studies with different characteristics according to the following categorical: patient source, publication year, age, proportion of male, economic circles, geographical distribution, and the combination of economic circles and geographical distribution (since the economic circles were only divided in mainland China, the relevant analyses were conducted based on researches in mainland China.). Subsequently, meta-regression analysis (Restricted Estimation Maximum Likelihood, REML) was performed to examine potential sources of heterogeneity (22). β statistics determined the results of moderator analyses, its *P*-value determined significance of the covariates and *R*^2^ indicated the proportion of true heterogeneity that could be explained by the covariate or covariates. Covariates of univariate meta-regression analysis included patient source, publication year, economic circle, geographical distribution, age, proportion of male, disease course, proportion of married patients, proportion of patients with family history of mental disorders, GDP per capita, unemployment rate, total burden coefficient, illiteracy rate, beds per 1,000 population in health institutions. Multivariate meta-regression analysis was conducted according to the following criteria: first, factors theoretically associated with violence-TO; second, variables with *P* < 0.10 in univariate meta-regression analysis; third, removing variables with collinearity; finally, *R*^2^ was considered.

Sensitivity analysis was done in main analysis, subgroup analysis and meta-regression analysis, by comparing results of eligible studies and those of high-quality studies. Significant level was set as two-sided and *P* < 0.05.

## Results

### Study characteristics

**FIGURE 1:**
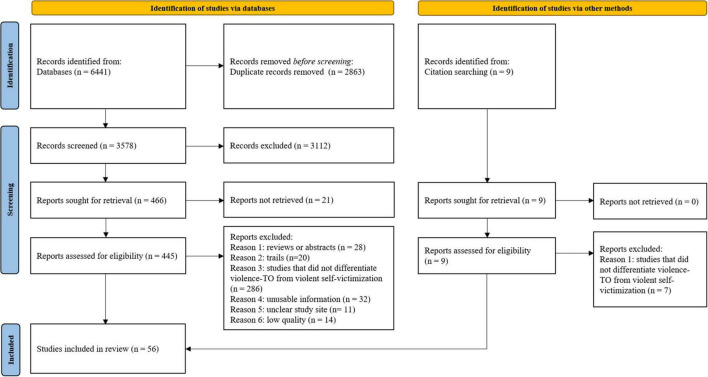
Study selection.

Only 56 unique studies between 1996 and 2022 were eligible for this meta-analysis, with 35, 14, 17, and 4 studies on type I to type IV, respectively. Please see the study selection flowchart in Figure 1. The characteristics of studies were shown in Table 1. 34 articles studied on inpatients and 22 studied on non-inpatients, with a total of 52,125 individuals with schizophrenia being included. According to the NOS quality assessment, there were 34 studies considered to be high-quality. Quality of studies was shown in Supplementary Table 3.

**TABLE 1:** Characteristics of studies included in the meta-analysis.

References	Research time	Study site	Study type	Coastal area	Economic circle	Diagnostic criteria	Patient source	Sample size (*N*)	Male (*N*)	Age (year)	Type of violence	Quality assessment
Sha et al. (23)	NA	Yangzhou, Jiangsu	CS	No	Yes	CCMD-2	Inpatient	80	80	45.16	I	7
Gan and Lv (24)	NA	Jinan, Shandong	CS	No	No	CCMD-2	Inpatient	125	92	28.20	I	7
Zhou (25)	NA	Nanchong, Sichuan	CS	No	No	CCMD-2	Inpatient	141	NA	NA	II	4
Cui et al. (26)	1994.5–1996.4	Jinan, Shandong	CS	No	No	CCMD-2	Inpatient	1,030	607	31.90	III	7
Ma and Hu (27)	1990.6–1997.5	Jiamusi, Heilongjiang	CS	No	No	DSM-II	Inpatient	280	161	NA	II	5
Wang and Yan (28)	1996.8–2000.2	Zibo, Shandong	CS	No	No	CCMD-2	Inpatient	1,018	NA	NA	III	4
Lv et al. (29)	2001.3	Luoyang, Henan	CS	No	No	CCMD-2	Inpatient	188	143	34.24	I	7
Zhang et al. (30)	NA	Jining, Shandong	CS	No	No	CCMD-2	Inpatient	149	NA	NA	II	4
Li (31)	1999.6–1999.8	Xinxiang, Henan	CS	No	No	CCMD-2	Inpatient	58	0	30.79	I, II, III	7
Li and Zhou (32)	1970.9–2002.1	Xiamen, Fujian	CS	Yes	No	CCMD-3	Inpatient	386	303	42.40	III, IV	7
Chen and Huang (33)	2003.9–2003.11	Longquan, Guangxi	CS	No	No	CCMD-3	Inpatient	147	147	30.60	I, III	5
Zhu and Wang (34)	1998.1–2003.1	Hohhot, Inner Mongolia	CS	No	No	CCMD-3	Inpatient	215	136	28.00	I	6
Gao et al. (35)	2004.1–2004.12	Beijing	CS	No	Yes	CCMD-3	Inpatient	106	106	30.60	III	7
Li et al. (36)	2005.1–2005.10	Beijing	CS	No	Yes	CCMD-3	Inpatient	148	148	NA	II, III	4
Zhang and Li (37)	NA	Beijing	CS	No	Yes	CCMD-3	Inpatient	92	77	42.58	IV	4
Xiong et al. (38)	2005.7–2006.11	Zigong, Sichuan	CS	No	No	CCMD-3	Inpatient	158	98	36.51	I, II	7
Guo (39)	2003.1–2003.12	Luoyang, Henan	CS	No	No	CCMD-3	Inpatient	193	102	25.35	I	6
Ran et al. (40)^†^	1994.1–2008.1	Chengdu, Sichuan	C	No	No	ICD-10	Non-inpatient	489	224	44.79	I, III, IV	7
Ou et al. (41)	NA	Minqing, Fujian	CS	No	No	CCMD-3	Non-inpatient	1,067	NA	NA	I	7
Sun et al. (42)	2006.1–2008.12	Yantai, Shandong	CS	Yes	No	CCMD-3	Inpatient	195	121	44.00	III	6
Gong et al. (43)	2010.3–2010.5	Guangzhou, Guangdong	CS	Yes	Yes	CCMD-3	Non-inpatient	636	NA	NA	I	7
Wang et al. (44)	NA	Huizhou, Guangzhou	CS	Yes	Yes	CCMD-3	Non-inpatient	919	NA	NA	I	7
Wei et al. (45)	2009.11–2011.3	Guangzhou, Guangdong	CS	Yes	Yes	CCMD-3	Inpatient	40	25	24.13	III	7
Xiong et al. (46)	2006.10–2009.10	Chengdu, Sichuan	CS	No	No	ICD-10	Non-inpatient	307	227	36.20	I	7
Yang (47)	2009.1–2009.12	Luoyang, Henan	CS	No	No	CCMD-3	Inpatient	196	104	32.18	I	5
Pan et al. (48)	2002.1–2011.1	Liaocheng, Shandong	CS	No	No	CCMD-3	Inpatient	500	269	NA	I, IV	6
Liang and Liu (49)	NA	Nanning, Guangxi	CS	No	No	CCMD-3	Non-inpatient	601	NA	NA	I	7
Zhang et al. (50)	2011.10–2012.3	Shantou, Guangdong	CS	Yes	No	CCMD-3	Non-inpatient	426	264	34.48	I	7
Song et al. (51)	2011.9–2012.11	Foshan, Guangdong	CS	No	Yes	CCMD-3	Non-inpatient	2,333	NA	NA	I	7
Liu et al. (52)	2012.3–2012.9	Yinchuan, Ningxia	CS	No	No	ICD-10	Inpatient	194	115	NA	I	5
Chen et al. (53)^†^	NA	Gaoxiong, Taiwan	C	Yes	NA	DSM-IV	Inpatient	107	33	33.40	I, II	9
Xia (54)	2010.1–2013.6	Zhenjiang, Jiangsu	CS	No	Yes	CCMD-3	Inpatient	48	35	35.12	II	4
Lang et al. (55)	2010.1–2013.1	Shizuishan, Ningxia	CS	No	No	CCMD-3	Non-inpatient	380	NA	NA	I	6
Zhang et al. (56)	NA	Kunming, Yunnan	CS	No	No	ICD-10	Non-inpatient	548	NA	NA	I	7
Wu (57)	2011.9–2014.12	Liaocheng, Shandong	CS	No	No	ICD-10	Inpatient	158	112	33.87	II, III	5
Li (58)	2011.1–2011.12	Zhenjiang, Jiangsu	CS	No	Yes	CCMD-3	Inpatient	138	74	NA	II	5
Qiao and Di (59)	NA	Lanzhou, Gansu	CS	No	No	CCMD-3	Non-inpatient	3,945	NA	NA	I	7
Zheng (60)	2008.1–2014.1	Nanning, Guangxi	CS	No	No	CCMD-3	Inpatient	1,283	NA	NA	I	7
Hu and He (61)	2014.3–2016.3	Wuhan, Hubei	CS	No	No	ICD-10	Non-inpatient	571	NA	NA	I	5
Yu et al. (62)	2010.1	Shaoxing, Zhejiang	CS	Yes	Yes	CCMD-3	Non-inpatient	766	NA	NA	I	7
Ding et al. (63)	2016.12	Beijing	CS	No	Yes	CCMD-3	Non-inpatient	1,740	NA	NA	I	7
Yuan et al. (64)	2008.1–2017.12	Yichang, Hubei	CS	No	No	ICD-10	inpatient	587	NA	NA	III	7
Huang et al. (65)	2008.1–2014.12	Nanning, Guangxi	CS	No	No	CCMD-3	Non-inpatient	1,637	NA	NA	I	7
Yang et al. (66)	2018.3–2018.7	Zhanjiang, Guangzhou	CS	Yes	No	ICD-10	Inpatient	226	116	35.12	I	7
Wang et al. (67)	NA	Gaoxiong, Taiwan	CS	Yes	NA	DSM-IV	Inpatient	33	16	49.50	I	7
Sun and Zhou (68)	2018.10–2019.10	Xinxiang, Henan	CS	No	No	ICD-10	Inpatient	84	46	NA	I, II, III	5
Li et al. (69)	2017.1–2019.12	Wuxi, Jiangsu	CS	No	Yes	CCMD-3	Non-inpatient	15,233	NA	NA	I	7
Chen et al. (70)	2018.10–2019.8	Gaoxiong, Taiwan	CS	Yes	NA	DSM-V	Non-inpatient	78	28	42.59	II	7
Zhao (71)	2016.12–2019.12	Liaoyang, Liaoning	CS	No	No	CCMD-3	Inpatient	201	125	36.02	III	7
Sun et al. (72)	NA	NA	CS	NA	NA	DSM-III	Non-inpatient	7711	4491	NA	III	7
Li (73)	2013.01–2020.01	Yangjiang, Guangdong	CS	Yes	No	CCMD-3	Inpatient	1180	NA	NA	I	5
Hu et al. (74)	2021.01–2021.12	Ali, Tibet	CS	No	No	ICD-10	Non-inpatient	59	NA	NA	II	5
Liang et al. (75)	2016.03–2019.10	Hainan	CS	Yes	No	CCMD-3	non-inpatient	98	NA	NA	I	5
Long et al. (76)	2019.03–2019.09	Changsha, Hunan	CS	No	No	CCMD-3	Non-inpatient	400	200	46.87	III	7
Pan et al. (77)	2019.04–2019.07	Beijing	CS	No	Yes	ICD-10	Non-inpatient	2,100	940	39.80	I	7
Yu et al. (78)	2021.03–2021.08	Hefei, Anhui	CS	No	No	DSM-V	Inpatient	397	397	39.86	III	7

*NA, Not Available; C, Cohort study; CS, Cross-Section study.

^†^We used the baseline data in these two studies.

### Main analysis

**FIGURE 2:**
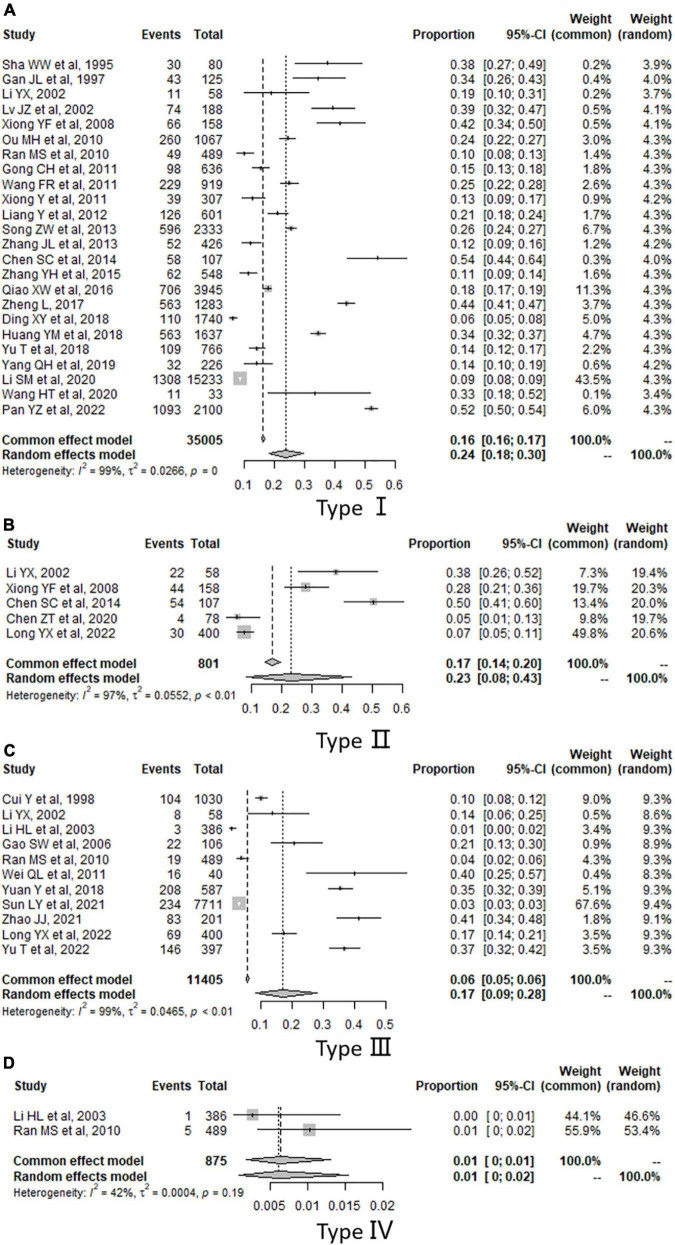
Forest plot of the prevalence of violence. **(A)** Results of type I. **(B)** Results of type II. **(C)** Results of type III. **(D)** Results of type IV.

The pooled estimates in high-quality studies showed that the prevalence of each type of violence-TO from high to low was type I (23.83%, 95% CI: 18.38–29.75%), type II (23.16%, 95% CI: 8.04–42.97%), type III (17.19%, 95% CI: 8.52–28.04%) and type IV (0.62%, 95% CI: 0.08–1.54%) (Table 2). Forest plots are shown in Figure 2.

**TABLE 2:** Pooled estimate of prevalence of violence.

	Eligible studies					High-quality studies				
Type of violence	Number of data points	Pooled prevalence (%, 95% CI)	*Q*	*Q*-test *P*-value	*I*^2^ (%)	Number of data points	Pooled prevalence (%, 95% CI)	*Q*	*Q*-test *P*-value	*I*^2^ (%)
I	35	26.44 (20.62–32.68)	7317.23	<0.01	99.50	24	23.83 (18.38–29.75)	3579.51	<0.01	99.40
II	14	31.17 (21.70–41.49)	284.46	<0.01	95.40	5	23.16 (8.04–42.97)	126.59	<0.01	96.80
III	17	16.21 (9.57–24.16)	1257.77	<0.01	98.70	11	17.19 (8.52–28.04)	1117.84	<0.01	99.10
Iv	4	2.33 (0.31–5.90)	33.05	<0.01	90.90	2	0.62 (0.08–1.54)	1.72	0.19	41.90

Sensitivity analysis was done by compared with the results of all eligible studies and those of high-quality studies, which showed that the pooled estimates of prevalence in all types of violence-TO not changed significantly, which meant our estimates were relatively stable (Table 2). We also did analysis by removing each study individually, and results were shown in Supplementary Tables 4–7, which also reflected the robustness of our analysis.

The publication bias was tested in type I and type III. Egger linear regression test showed that there existed the publication bias in type I (*t* = 2.18, *P* = 0.04) and type III (*t* = 2.74, *P* = 0.02). The “Trim and Fill” method was used to adjust the bias, and the results of type III didn’t change obviously, which meant that the bias had little influence on the pooled estimation, but the results of type I varied significantly, which indicated that it was influenced by the bias (Supplementary Table 8). Funnel plots are shown in Supplementary Figures 1, 2.

**FIGURE 3:**
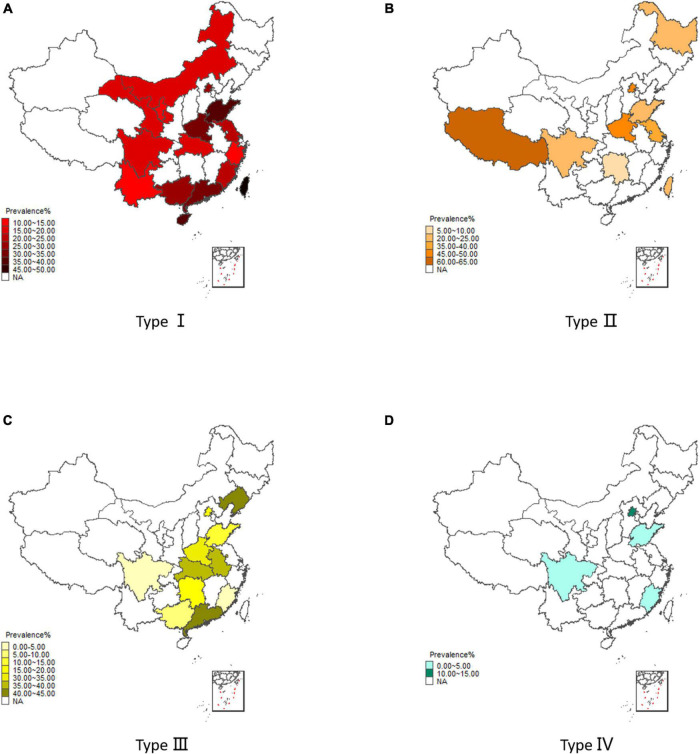
Regional distribution for prevalence of violence. **(A)** Results of type I. **(B)** Results of type II. **(C)** Results of type III. **(D)** Results of type IV.

The pooled estimates of each type of violence in each province were calculated (Supplementary Table 9), and the statistical map was shown in Figure 3. The top three regions with the highest prevalence of type I to type III were as follows: type I: Taiwan (45.00%), Hainan Province (39.80%), and Shandong Province (39.12%); type II: Tibet (61.02%), Henan Province (49.63%), and Beijing City (47.97%); type III: Liaoning Province (41.29%), Guangdong Province (40.00%), and Anhui Province (36.78%). There were only four articles included in type IV and the highest prevalence was in Beijing City (14.13%).

### Subgroup analysis and meta-regression analysis

Then the subgroup analysis and the meta-regression analysis were applied to examine potential sources of heterogeneity of type I to type III (type IV was excluded due to inadequate studies).

The results of subgroup analysis showed that: first, the prevalence of type I and type II was higher among inpatients than non-inpatients; second, the prevalence of type II was higher among patients aged under 40; finally, in the analyses of spatial distribution, when grouped by economic circles or geographic distribution separately, the differences among groups were not statistically significant. When grouped by economic circles combined with geographic distribution, the prevalence of type I was higher among subjects in the inland than in the coastal non-economic zone, while the prevalence of type III was highest in the coastal economic zone, followed by the inland region and lowest in the coastal non-economic zone (Supplementary Table 10).

Univariate meta-regression analysis was first conducted in type I to type III, which was shown in Supplementary Tables 12, 13. In type I, patient source (β = 0.19, *P* < 0.01) and total burden coefficient (β = 1.00, *P* = 0.02) were statistically significant; in type II, patient source (β = 0.41, *P* < 0.01) and age (β = 0.41, *P* < 0.01) were statistically significant; in type III, age (β = 0.29, *P* = 0.01), disease course (β = 0.37, *P* = 0.02), GDP per capita (β = 0.07, *P* < 0.01) and unemployment rate (β = –22.83, *P* < 0.01) were statistically significant. Multivariate meta-regression analysis was subsequently performed, which was shown in Table 3. The variables included in the model for type I were patient source (β = 0.15, *P* < 0.01) and total burden coefficient (β = 0.60, *P* = 0.13), which could explain 42.03% of the study heterogeneity, with higher prevalence in inpatients. In type II, the variables included were patient source (β = 0.47, *P* < 0.01) and proportion of male (β = 0.19, *P* < 0.01), which could explain 99.23% heterogeneity, with higher prevalence in female inpatients. The variables included in type III were age (β = 0.25, *P* < 0.01) and GDP per capita (β = 0.05, *P* = 0.01), which could explain 87.40% heterogeneity, with a higher prevalence of violence-TO among subjects aged under 40 and with higher GDP per capita.

**TABLE 3:** The results of multivariate meta-regression analysis (high-quality studies).

Type of violence	Moderators	Number of data points	Coefficient (β)	Coefficient *P*-value	*R*^2^ (%)
I	Patient source (inpatient/non-inpatient)	20	0.15	<0.01	42.03
	Total burden coefficient		0.60	0.13	
II	Patient source (inpatient/non-inpatient)	5	0.47	<0.01	99.23
	Proportion of male patients (≤ 0.5/ > 0.5)		0.19	<0.01	
III	Age (≤ 40/ > 40)	6	0.25	<0.01	87.40
	GDP per capita ($10,000)		0.05	0.01	

Results of sensitivity analysis showed that the estimates in the subgroup analysis (Supplementary Tables 10, 11) and coefficient in meta-regression analysis (Table 3 and Supplementary Tables 13–15) didn’t change obviously, which reflected the robustness of our analysis.

## Discussion

This study was the first systematic review and meta-analysis on the prevalence of violence-TO among schizophrenics in China, providing a comprehensive picture of the prevalence of different types of violence-TO in schizophrenia patients from different sources, and combining results of the subgroup analysis and the meta-regression to explore the heterogeneity in depth. We found that the prevalence of the four types of violence-TO was different, and the prevalence of inpatients was higher in most types of violence-TO. Heterogeneity sources varied among the types, and in addition to the individual characteristics including patient source, proportion of male and age, regional-level indicators such as spatial distribution and GDP per capita could also explain part of the heterogeneity, which might affect the prevalence of violence-TO.

### Definitions and classifications of violence

The first focus is about the considerations regarding the definition and classification of violence. Some researchers, represented by Whiting et al., argued that violence could not be defined or measured in a simple way at present (79). First, many terms were related to violence including aggression, agitation, offense, risk behavior and so on. Second, various instruments are used for measuring violence. As mentioned above, there are three forms of violence experienced by people with mental disorders (8). Some definitions or scales did not distinguish victims of violence and collectively called self-victimization and violence-TO “violence,” such as MOAS. Among various definitions after distinguishing the victims of violence, such as the violence-TO, which the study focused on, disagreement still existed, mainly on whether harmless behaviors, like verbal attack, were violence-TO. For example, the Chinese legal system considers violence-TO to be an act that targets the person or property and causes great damage to the physical or mental health, life or property of the victim, and directly endangers the life, health of the person (80). Behavioral science considers violence-TO as an act of harming or attempting to harm the psychological or physical state of other individuals or destroying other targets (81).

Previous systematic reviews on the prevalence of violence in subjects with mental disorders or schizophrenia had some limitations. First, they focused on a single act of violence. In 2014, Zhou et al. (13) conducted a systematic review on the prevalence of violence-TO in acute psychiatric inpatients from 12 high-income countries, with 23,972 patients suffering from mental disease, including schizophrenia, were included, which focused only on “physically harmful behavior.” Second, the target of violence was not differentiated. In 2019, Zhou et al. (13) estimated the prevalence of violence in terms of Chinese schizophrenia inpatients with a total of 3,941 patients involved. In 2020, a systematic review was conducted by Li et al. (15) on the prevalence of violence in subjects with schizophrenia worldwide with 3,929 individuals involved. These two studies both used “MOAS ≥ 3” as a criterion for inclusion of outcomes.

After systematically retrieving all the resources about the definition of violence-TO, this study referred to a relatively more comprehensive definition of violence-TO published by Douglas et al. (9) (see Background). Its strength lied in the review of the definitions or measures of violence-TO in 204 high-quality studies and the classification of violence-TO, which compensated for the incomprehensive and imprecise definitions of violence-TO. The result of this study showed that the prevalence of type I was 23.83%. A study conducted by Bulgari et al. in Italy included 87 inpatients with schizophrenia (82). According to the data from the Department of Mental Health, the prevalence of previous violence-TO at baseline was 36.72% in these patients, higher than that of this study. This might be due to the fact that the study included only inpatients and some of them received compulsory treatment because of extreme behaviors (e.g., violence). However, both studies suggested that previous violence was more common in individuals with schizophrenia and that subjects with history of violence were at a greater risk of violence-TO in the future (83), which implied that these subjects required more stringent management measures. In terms of type II and type III, some studies found that the prevalence of verbal attack might be three times higher than that of physical harm, and the prevalence of homicide might be low (84), which was similar to the result of our study. Type II was more common in Chinese schizophrenics (23.16%), followed by type III (17.19%), and the prevalence of these two types of violence-TO was lower in China compared to global individuals with psychiatric disorders or schizophrenia. Li et al. found that the prevalence of verbal attack and physical harm in global individuals with schizophrenia was about 42.6 and 23.8%, respectively (15). This might also be related to the focus on inpatients in the study above. Type IV, which could cause grave consequences, was relatively rare, and the prevalence of this type of violence-TO in China (0.62%) in our study was much lower than the global level (2.30%) (85). This might be due to the fact that only two high-quality studies on type IV were involved and the results were underrepresented. In summary, compared with studies in other countries and regions, the prevalence of all types of violence-TO was generally low in China. In addition to the reasons mentioned above, it might be related to the underreporting of people with schizophrenia and their violence-TO behavior. Liu et al. (86) concluded that multiple systems existed in the registration of violence-TO for people with schizophrenia in China, such as medical care and legislation, but data could not be shared among systems so that exact data could not be obtained. The study conducted by Liu et al. (87) also showed that there were certain problems, including under-reporting, missing values, and errors, in the registration of violence among people with severe mental disorder (SMD) in China, and data quality might affect the accuracy of the results to some extent.

### Patient sources

Second, our study focused on patients from different sources. Zhou et al. (13) found the prevalence of violence-TO in acute psychiatric inpatients with mental disorders was 17%. Zhou et al. (13) found the prevalence of violence in Chinese inpatients with schizophrenia was 35.4%. The systematic review conducted by Li et al. (15) didn’t restrict the patient source, of which the pooled estimate of prevalence of violence in worldwide schizophrenics was 33.3%. In a subgroup analysis by nationality, the pooled estimate was 50.7% in Chinese, but there were only three articles included in this subgroup, and all studied on inpatients. The result of our study showed that the prevalence of type I to type IV was 23.83, 23.16, 17.19, 0.62%, respectively, generally lower than those in the above studies. This might be due to the fact that most of these studies focused on inpatients with schizophrenia or mental disorders, hence the prevalence could not be extrapolated to all individuals with schizophrenia including those in the community (13). In contrast, we calculated the prevalence of violence-TO in Chinese schizophrenics by including both inpatients and non-inpatients and further conducted subgroup analysis based on the source of patients to explore the prevalence of violence-TO in subjects from different sources. Therefore, the information provided in this study was relatively more comprehensive and detailed in its consideration of the subjects studied.

### Sources of heterogeneity

The last point was about the investigation into sources of heterogeneity. Previous systematic reviews were highly heterogeneous. For example, Zhou et al. (13) found that the overall heterogeneity test result of *I*^2^ was 98.2%, but no obvious sources of heterogeneity were detected in the subgroup analysis. Without reporting the overall heterogeneity analysis result, Zhou et al. (13) solely depended on the subgroup analysis to search sources of heterogeneity, and found that the *I*^2^ of each subgroup ranged from 71.6 to 94.5% while at the same time no significant sources of heterogeneity were detected. An overall heterogeneity analysis result with *I*^2^ of 98.07% was reported by Li et al. (15), who revealed that study site, time, and quality assessment might contribute to heterogeneity by subgroup analysis and univariate meta-regression analysis, yet no further multivariate meta-regression analysis was conducted. Similar to previous studies, there was moderate to high heterogeneity (41.90–99.40%) in the prevalence of the four types of violence-TO in our study. Therefore, we investigated heterogeneity by combining results of subgroup analysis and those of both univariate and multivariate meta-regression analysis. Meanwhile, besides individual factors, we included potentially relevant regional-level indicators from the statistical year book for the analyses. The result showed that the reasons for heterogeneity varied across the different types of violence-TO, and the possible sources of heterogeneity including patient sources, proportion of male, age, spatial distribution and GDP per capita.

First of all, patient sources showed a higher prevalence of inpatients than non-inpatients in most types of violence-TO. Subgroup analysis demonstrated a higher prevalence of inpatients than non-inpatients, and multivariable meta-regression analysis showed patient source β of 0.15 in type I and β of 0.47 in type II. For type III, with a trend of higher prevalence in inpatients, the difference was not significant, but the result was instructive in practice. The results were similar to those in previous studies. Such as Li et al. (15), the results were approximately 45.1% for inpatients while only 4.6% for community subjects. Bobes et al. showed that inpatients’ conditions were usually unstable, and most had limited cognition of their illness. Besides, inpatients were more severely affected by positive symptoms (e.g., hallucinations, delusions). It may explain why they were more prone to violent behavior (88).

Second, this study showed that lower male proportion was associated with higher prevalence of type II violence-TO, which was contrary to some previous studies. Males with schizophrenia are more likely to act violence generally (4, 7, 13, 15, 89). Such as an investigation on the crime rate in schizophrenics in Germany during 1973–2004, conducted by Soyka et al. (89), it reported that the rate was 7% in males while only 1.4% in females. But all studies mentioned above didn’t report the prevalence of different types of violence-TO by gender. On the one hand, due to the fact that females are weaker than males in physical strength, they rarely acted serious forms of violence-TO (like type III and IV). For example, research on intimate partner violence by Thornton et al., found that violence-TO in females was more likely to be pushing, throwing objects and so on, while other harmful violence-TO, such as violent crime, has a higher prevalence in males (90). Probably for similar reasons, the result showed a trend that higher prevalence of type II might be associated with lower male proportion. On the other hand, limited by the number of available studies, there were only 5 data points included in the model, which showed poor extrapolation to some extent. The association between gender and different types of violence-TO needs to be explored after more high-quality literature available in the future.

Third, results of subgroup analysis and meta-regression analysis showed that the prevalence of violence-TO in subjects aged under 40 was higher than that in those aged above 40. This was consistent with the findings of previous studies, such as Liu et al. (87), who analyzed 121,830 individuals with severe mental disorders (SMD) in Sichuan province. They found that middle age (45–59 years old) had no significant association with violence, while adolescent (15–24 years old) was a risk factor for violence compared to young adults (25–44 years old) (IRR = 1.14). By contrast, the elder (≥ 60 years old) was a protective factor from violence (IRR = 0.88). After analyzing 78 schizophrenics aged from 20 to 64 years old, Chen et al. (70) also found a negative correlation between age and physical harm (β = –0.02). The above may be related to the fact that the physical function of the patient decreases with age and therefore the prevalence of violence-TO is lower in older people (33).

Fourth, in the subgroup analysis of type I and III, we found that the prevalence of violence-TO varied between regions, showing greater variation in coastal areas and less variation in inland zones. The following was the comparison of the prevalence in different subgroups in the analysis of the combination of economic circles and geographical areas. China had huge economic and cultural differences among regions. By combining the rapidly developing economic cities, the national development plan designed these cities to be economic circles, which had been constructed as the Pearl River Delta Economic Circle (91), the Yangtze River Delta Economic Circle (92) and the Beijing-Tianjin-Hebei Economic Circle (93). As regards the results from type I and III, the lower prevalence was found in the coastal non-economic zone (type I: 12.86%, type III: 0.78%) while the higher prevalence was found in inland regions (type I: 24.44%, type III: 20.88%), and type III had the highest prevalence of 40.00% in coastal economic zones. Two possible reasons might be accountable: first, it might be affected by the detection rate of violence-TO because not all regions in China have established national management systems for SMD. Even though the legislation had some recordings, this information could not be shared among different departments (86). Second, Zhen-ye Li’s study on the happiness index of coastal cities in China indicated that it was mainly composed of two elements: the utility of economic gains and the environmental utility of public wellbeing. Although the former one was higher in the coastal economic zones, the latter one was greater in the coastal non-economic zones, which had an appropriate population density and a better living environment for their residents, who could enjoy more public resources (94). Therefore, the life quality of schizophrenics in the coastal non-economic zones was better than that in the coastal economic zones, and this might contribute to their lower prevalence of violence-TO.

Last but not least, GDP per capita, as a regional-level economic indicator, demonstrated a positive correlation with the prevalence of type III in this study, which was contrary to previous results. The study by Swanson et al. indicated that economic hardship and poor living conditions were prospective predictors of violence in schizophrenics (95). Yet the indicators in the above study were mostly the individual-level economic status, rather than the regional economic conditions where the subjects lived. When investigating influencing factors of violence among rural people with SMD in Sichuan province in China, Liu et al. (87) also included regional-level economic indicators such as the annual net income of rural residents per capita (ANIPC) in the research, and they found a decrease in ANIPC (β = –8.04) which led to an increase in violence. Since Saxena et al. suggested that socioeconomic disadvantage might lead to adverse outcomes of mental disorders (96), we speculated that there were two possible reasons for our results. First, it might relate to the detection rate as well. In addition, data quality problems such as under-reporting, and errors, were prevalent in the registration system of patients’ violence-TO with SMD (86). In contrast, the management system was more advanced in developed areas, so the detection rate of violence-TO in these areas was higher. In undeveloped areas, since the relevant institutions failed to find the violence-TO of schizophrenics in time and record it (97), the prevalence of violence-TO in developed areas, instead, was presented to be much higher. Second, Liu et al. (87) only included individuals with SMD in some rural areas from western China, while this study focused on individuals with schizophrenia in China as a whole. In all, different study regions and subjects might lead to certain heterogeneity in the prevalence of violence-TO.

### Limitations

In addition to the above findings, this study still has some limitations, mainly including the following points. First, due to the objective limitations of existing original studies, the number of articles included in this study was limited. This resulted in a certain degree of under-representation. Second, the number of data points included in the multivariable meta-regression analysis were limited, thus, more meaningful covariates failed to be explored, which also led to insufficient power of a test in some multivariable regression models. Third, previous studies identified possible sources of heterogeneity, especially some biological or clinical aspects, such as testosterone level, substance abuse, neuroimages, antipsychotic treatment, violence history, and so on (5, 13–15). But most of the information had not been reported in the original studies, leading to no further research into the known sources of heterogeneity. Fourth, corresponding to earlier studies, some regional-level indicators extracted from statistical year books were missing, which led to data unavailable for some studies as well. And these factors associated with violence-TO in schizophrenics can be further explored when more studies are carried out. Fifth, due to data availability, this study chose the publication year instead of the research time in terms of heterogeneity investigation, which might result in some bias. At last, although we summarized high-quality studies separately, bias in type I and type III did exist, and that in type I could not be adjusted by the “Trim and Fill” method. Both outcome measure bias with unclear definitions of violence-TO and patient selection bias in the original study might be accountable for this, and the “Trim and Fill” method failed to identify them (21). It might be also affected by the under detection of related literatures, such as the unpublished or articles published other than Chinese or English. Therefore, the type I pooled estimate had limited validity and the findings should be reviewed with caution.

## Conclusion

In summary, the conclusions of this study are as follows. First, schizophrenics may act many different types of violence-TO, which has diverse prevalence and influencing factors. It implies that work on violence-TO prevention and control cannot be generalized, and different management measures should be adopted for different types of violence-TO. Second, individual-level factors that may be associated with the prevalence, can help to identify patients with high risk on different violence-TO behavior. Third, this study has found that regional-level indicators may affect the violence-TO of schizophrenics as well. Yet, few studies have examined the association among these factors and violence-TO in individuals with schizophrenia. Results regarding the significant variations in the prevalence of violence-TO between regions may advocate the health authorities to make decisions based on resource distribution with clear localized targets (87). The prevention and treatment of violence-TO among subjects with schizophrenia requires the cooperation of multiple departments. Therefore, carrying out such research contributes to helping national departments to formulate targeted policy measures in the macro level and to achieving the primary prevention, so as to further promoting the development of public health care in China and around the world.

## Data availability statement

The original contributions presented in the study are included in the article/Supplementary Material, further inquiries can be directed to the corresponding author/s.

## Author contributions

YG, XY, YuL, and XL had full access to all data in the study and take responsibility for the integrity of the data and the accuracy of the data analysis. YuL and XL conceived and designed the study. YG, XY, and DW selected the articles. RF, YiL, and RW extracted the data. YG and HX analyzed the data. YG, YuL, and XL interpreted the data and wrote the first draft of the manuscript. All authors contributed to critical revision of the report for important intellectual content, read, and met the ICMJE criteria for authorship and agreed with the results and conclusions of this article.

## Conflict of interest

The authors declare that the research was conducted in the absence of any commercial or financial relationships that could be construed as a potential conflict of interest.

## Publisher’s note

All claims expressed in this article are solely those of the authors and do not necessarily represent those of their affiliated organizations, or those of the publisher, the editors and the reviewers. Any product that may be evaluated in this article, or claim that may be made by its manufacturer, is not guaranteed or endorsed by the publisher.
