# Supplementary material for: Prevalence of violence to others among individuals with schizophrenia in China: A systematic review and meta-analysis
Source: Front Psychiatry. 2022 Jul 22;13:939329. doi: 10.3389/fpsyt.2022.939329 (PMC9354073; doi:10.3389/fpsyt.2022.939329)
Supplement: Supplementary file 1 [file Data_Sheet_1.docx]

**Supplementary appendix**

**TABLE A1|** PRISMA checklist

| **Section and Topic** | **Item** | **Checklist item** | **Location where item is reported** |
| --- | --- | --- | --- |
| **TITLE** | | |  |
| Title | 1 | Identify the report as a systematic review. | 1 |
| **ABSTRACT** | | |  |
| Abstract | 2 | See the PRISMA 2020 for Abstracts checklist. | 1-2 |
| **INTRODUCTION** | | |  |
| Rationale | 3 | Describe the rationale for the review in the context of existing knowledge. | 2-3 |
| Objectives | 4 | Provide an explicit statement of the objective(s) or question(s) the review addresses. | 3 |
| **METHODS** | | |  |
| Eligibility criteria | 5 | Specify the inclusion and exclusion criteria for the review and how studies were grouped for the syntheses. | 4 |
| Information sources | 6 | Specify all databases, registers, websites, organisations, reference lists and other sources searched or consulted to identify studies. Specify the date when each source was last searched or consulted. | 3 |
| Search strategy | 7 | Present the full search strategies for all databases, registers and websites, including any filters and limits used. | 3; Appendix TABLE A2 |
| Selection process | 8 | Specify the methods used to decide whether a study met the inclusion criteria of the review, including how many reviewers screened each record and each report retrieved, whether they worked independently, and if applicable, details of automation tools used in the process. | 4 |
| Data collection process | 9 | Specify the methods used to collect data from reports, including how many reviewers collected data from each report, whether they worked independently, any processes for obtaining or confirming data from study investigators, and if applicable, details of automation tools used in the process. | 4 |
| Data items | 10a | List and define all outcomes for which data were sought. Specify whether all results that were compatible with each outcome domain in each study were sought (e.g. for all measures, time points, analyses), and if not, the methods used to decide which results to collect. | 4 |
|  | 10b | List and define all other variables for which data were sought (e.g. participant and intervention characteristics, funding sources). Describe any assumptions made about any missing or unclear information. | 4 |
| Study risk of bias assessment | 11 | Specify the methods used to assess risk of bias in the included studies, including details of the tool(s) used, how many reviewers assessed each study and whether they worked independently, and if applicable, details of automation tools used in the process. | 4 |
| Effect measures | 12 | Specify for each outcome the effect measure(s) (e.g. risk ratio, mean difference) used in the synthesis or presentation of results. | 4-5 |
| Synthesis methods | 13a | Describe the processes used to decide which studies were eligible for each synthesis (e.g. tabulating the study intervention characteristics and comparing against the planned groups for each synthesis (item #5)). | 5 |
|  | 13b | Describe any methods required to prepare the data for presentation or synthesis, such as handling of missing summary statistics, or data conversions. | 5 |
|  | 13c | Describe any methods used to tabulate or visually display results of individual studies and syntheses. | 5 |
|  | 13d | Describe any methods used to synthesize results and provide a rationale for the choice(s). If meta-analysis was performed, describe the model(s), method(s) to identify the presence and extent of statistical heterogeneity, and software package(s) used. | 5 |
|  | 13e | Describe any methods used to explore possible causes of heterogeneity among study results (e.g. subgroup analysis, meta-regression). | 5 |
|  | 13f | Describe any sensitivity analyses conducted to assess robustness of the synthesized results. | 5 |
| Reporting bias assessment | 14 | Describe any methods used to assess risk of bias due to missing results in a synthesis (arising from reporting biases). | 5 |
| Certainty assessment | 15 | Describe any methods used to assess certainty (or confidence) in the body of evidence for an outcome. | 5 |
| **RESULTS** | | |  |
| Study selection | 16a | Describe the results of the search and selection process, from the number of records identified in the search to the number of studies included in the review, ideally using a flow diagram. | FIGURE 1 |
|  | 16b | Cite studies that might appear to meet the inclusion criteria, but which were excluded, and explain why they were excluded. | FIGURE 1 |
| Study characteristics | 17 | Cite each included study and present its characteristics. | 5; TABLE 1 |
| Risk of bias in studies | 18 | Present assessments of risk of bias for each included study. | Appendix TABLE A3 |
| Results of individual studies | 19 | For all outcomes, present, for each study: (a) summary statistics for each group (where appropriate) and (b) an effect estimate and its precision (e.g. confidence/credible interval), ideally using structured tables or plots. | TABLE 1 |
| Results of syntheses | 20a | For each synthesis, briefly summarise the characteristics and risk of bias among contributing studies. | TABLE 1 |
|  | 20b | Present results of all statistical syntheses conducted. If meta-analysis was done, present for each the summary estimate and its precision (e.g. confidence/credible interval) and measures of statistical heterogeneity. If comparing groups, describe the direction of the effect. | 5-6; TABLE 2; FIGURE 2 |
|  | 20c | Present results of all investigations of possible causes of heterogeneity among study results. | 6-7; TABLE 3; Appendix TABLE A10-A15 |
|  | 20d | Present results of all sensitivity analyses conducted to assess the robustness of the synthesized results. | TABLE 2; Appendix TABLE A4-A7, A10-A15 |
| Reporting biases | 21 | Present assessments of risk of bias due to missing results (arising from reporting biases) for each synthesis assessed. | Appendix TABLE A8; Appendix FIGURE A1-A2 |
| Certainty of evidence | 22 | Present assessments of certainty (or confidence) in the body of evidence for each outcome assessed. | 5-6; TABLE 2 |
| **DISCUSSION** | | |  |
| Discussion | 23a | Provide a general interpretation of the results in the context of other evidence. | 7 |
|  | 23b | Discuss any limitations of the evidence included in the review. | 11 |
|  | 23c | Discuss any limitations of the review processes used. | 11 |
|  | 23d | Discuss implications of the results for practice, policy, and future research. | 11 |
| **OTHER INFORMATION** | | |  |
| Registration and protocol | 24a | Provide registration information for the review, including register name and registration number, or state that the review was not registered. | 3 |
|  | 24b | Indicate where the review protocol can be accessed, or state that a protocol was not prepared. | 3 |
|  | 24c | Describe and explain any amendments to information provided at registration or in the protocol. | NA |
| Support | 25 | Describe sources of financial or non-financial support for the review, and the role of the funders or sponsors in the review. | 12 |
| Competing interests | 26 | Declare any competing interests of review authors. | 11 |
| Availability of data, code and other materials | 27 | Report which of the following are publicly available and where they can be found: template data collection forms; data extracted from included studies; data used for all analyses; analytic code; any other materials used in the review. | 12 |

**TABLE A2|** Search strategy and results

| Database | Full search strategy for each database | Citations |
| --- | --- | --- |
| CBM | #1 "精神分裂症"[常用字段:智能]  #2 "暴力行为"[常用字段:智能] OR "肇事肇祸"[常用字段:智能] OR "危险行为"[常用字段:智能] OR "攻击行为"[常用字段:智能] OR "凶杀行为"[常用字段:智能] OR "违法行为"[常用字段:智能] OR "犯罪行为"[常用字段:智能] OR "伤害行为"[常用字段:智能] OR "冲动行为"[常用字段:智能]  #3 (#2) AND (#1) | 1309 |
| CNKI | SU=('精神分裂症') AND SU=('暴力行为'+'肇事肇祸'+'危险行为'+'攻击行为'+'凶杀行为'+'违法行为'+'犯罪行为'+'伤害行为'+'冲动行为') | 1170 |
| Wangfang | 主题:(精神分裂症) and 主题:((暴力行为 or 肇事肇祸 or 危险行为 or 攻击行为 or 凶杀行为 or 违法行为 or 犯罪行为 or 伤害行为 or 冲动行为)) | 2188 |
| VIP | M=(精神分裂症) AND M=(暴力行为+肇事肇祸+危险行为+攻击行为+凶杀行为+违法行为+犯罪行为+伤害行为+冲动行为) | 763 |
| EBSCO | #1 SU schizophrenia*  #2 SU violence* OR SU dangerous behavior* OR SU illegal behavior* OR SU aggression* OR SU criminal behavior* OR SU injury* OR SU offensive behavior* OR SU homicidal behavior* OR SU Agitation*  #3 TX China OR TX Chinese  #4 S1 AND S2 AND S3 | 151 |
| Embase | #1 schizophrenia*.tw.  #2 (violence* or dangerous behavior* or illegal behavior* or aggression* or criminal behavior* or injury* or offensive behavior* or homicidal behavior* or Agitation*).tw.  #3 (China or Chinese).af.  #4 1 and 2 and 3 | 257 |
| Medline | #1 schizophrenia*.tw.  #2 (violence* or dangerous behavior* or illegal behavior* or aggression* or criminal behavior* or injury* or offensive behavior* or homicidal behavior* or Agitation*).tw.  #3 (China or Chinese).af.  #4 1 and 2 and 3 | 130 |
| PubMed | #1 Search: schizophrenia[Title/Abstract] Sort by: Most Recent  #2 Search: ((((((((violence*[Title/Abstract]) OR (dangerous behavior*[Title/Abstract])) OR (illegal behavior*[Title/Abstract])) OR (aggression*[Title/Abstract])) OR (criminal behavior*[Title/Abstract])) OR (injury*[Title/Abstract])) OR (offensive behavior*[Title/Abstract])) OR (homicidal behavior*[Title/Abstract])) OR (Agitation*[Title/Abstract]) Sort by: Most Recent  #3 Search: (China[Affiliation]) OR (Chinese[Affiliation]) Sort by: Most Recent  #4 Search: ((schizophrenia[Title/Abstract]) AND (((((((((violence*[Title/Abstract]) OR (dangerous behavior*[Title/Abstract])) OR (illegal behavior*[Title/Abstract])) OR (aggression*[Title/Abstract])) OR (criminal behavior*[Title/Abstract])) OR (injury*[Title/Abstract])) OR (offensive behavior*[Title/Abstract])) OR (homicidal behavior*[Title/Abstract])) OR (Agitation*[Title/Abstract]))) AND ((China[Affiliation]) OR (Chinese[Affiliation])) Sort by: Most Recent | 152 |
| Science Direct | (violence* OR dangerous behavior* OR illegal behavior* OR aggression* OR criminal behavior* OR injury* OR offensive behavior* OR homicidal behavior* OR agitation*) And (schizophrenia) And (China) | 98 |
| Web of Science | #1 TS=schizophrenia*  #2 TS=(violence* OR dangerous behavior* OR illegal behavior* OR aggression* OR criminal behavior* OR injury* OR offensive behavior* OR homicidal behavior* OR Agitation*)  #3 TS=(China OR Chinese)  #4 #1 AND #2 AND #3 | 223 |

**TABLE A3|** Quality assessment using Newcastle-Ottawa Scale

| **Cohort studies** | | | | | | | | | | | | | | | | | | | | |
| --- | --- | --- | --- | --- | --- | --- | --- | --- | --- | --- | --- | --- | --- | --- | --- | --- | --- | --- | --- | --- |
|  | Selection | | | | | | | | |  | Comparability | | |  | | Outcome | | |  |  |
| Study | Representativeness of the exposed cohort | | Selection of the non exposed cohort | | Ascertainment of exposure | | Demonstration that the outcome of interest was not present at start of the study | | |  | Comparability of cohorts on the basis of design or analysis | Assessment of outcome | |  | | Was follow‐up long enough for the outcome to occur? | | Adequacy of follow up of cohorts | Total stars | Good quality |
| Ran MS et al, 2010 | * | | * | |  | | * | | |  | ** |  | |  |  | * | | * | 7 | yes |
| Chen SC et al, 2014 | * | | * | | * | | * | | |  | ** | * | |  |  | * | | * | 9 | yes |
| **Cross-sectional studies** | | | | | | | | | | | | | | | | | | | | |
|  | Selection | | | | | | |  | Comparability | | | |  | | Outcome | | | |  |  |
| Study | | Representativeness of the exposed sample | | Selection of the non exposed sample | | Ascertainment of exposure | |  | Comparability of outcome groups on the basis of design or analysis | | | |  | | Assessment of outcome | | Statistical test is appropriate | | Total stars | Good quality |
| Sha WW et al, 1995 | | * | | * | | * | |  | ** | | | |  |  | * | | * | | 7 | yes |
| Gan JL et al, 1997 | | * | | * | | * | |  | ** | | | |  |  | * | | * | | 7 | yes |
| Zhou FJ, 1998 | |  | |  | | * | |  | * | | | |  |  | * | | * | | 4 | no |
| Cui Y et al, 1998 | | * | | * | | * | |  | ** | | | |  |  | * | | * | | 7 | yes |
| Ma CY et al, 1999 | | * | | * | | * | |  |  | | | |  |  | * | | * | | 5 | no |
| Wang J et al, 2001 | | * | | * | | * | |  |  | | | |  |  |  | | * | | 4 | no |
| Lv JZ et al, 2002 | | * | | * | | * | |  | ** | | | |  |  | * | | * | | 7 | yes |
| Zhang XH et al, 2002 | |  | |  | | * | |  | * | | | |  |  | * | | * | | 4 | no |
| Li YX, 2002 | | * | | * | | * | |  | ** | | | |  |  | * | | * | | 7 | yes |
| Li HL et al, 2003 | | * | | * | | * | |  | ** | | | |  |  | * | | * | | 7 | yes |
| Chen Q et al, 2004 | |  | |  | | * | |  | ** | | | |  |  | * | | * | | 5 | no |
| Zhu FY et al, 2006 | | * | | * | |  | |  | ** | | | |  |  | * | | * | | 6 | no |
| Gao SW et al, 2006 | | * | | * | | * | |  | ** | | | |  |  | * | | * | | 7 | yes |
| Li JH et al, 2007 | | * | | * | | * | |  |  | | | |  |  | * | |  | | 4 | no |
| Zhang CH et al, 2008 | |  | |  | | * | |  | * | | | |  |  | * | | * | | 4 | no |
| Xiong YF et al, 2008 | | * | | * | | * | |  | ** | | | |  |  | * | | * | | 7 | yes |
| Guo ZX, 2009 | | * | | * | | * | |  | * | | | |  |  | * | | * | | 6 | no |
| Ou MH et al, 2010 | | * | | * | | * | |  | ** | | | |  |  | * | | * | | 7 | yes |
| Sun ZX et al, 2010 | | * | | * | | * | |  | * | | | |  |  | * | | * | | 6 | no |
| Gong CH et al, 2011 | | * | | * | | * | |  | ** | | | |  |  | * | | * | | 7 | yes |
| Wang FR et al, 2011 | | * | | * | | * | |  | ** | | | |  |  | * | | * | | 7 | yes |
| Wei QL et al, 2011 | | * | | * | | * | |  | ** | | | |  |  | * | | * | | 7 | yes |
| Xiong Y et al, 2011 | | * | | * | | * | |  | ** | | | |  |  | * | | * | | 7 | yes |
| Yang YG et al, 2011 | | * | | * | | * | |  |  | | | |  |  | * | | * | | 5 | no |
| Pan SL et al, 2012 | | * | | * | | * | |  | * | | | |  |  | * | | * | | 6 | no |
| Liang Y et al, 2012 | | * | | * | | * | |  | ** | | | |  |  | * | | * | | 7 | yes |
| Zhang JL et al, 2013 | | * | | * | | * | |  | ** | | | |  |  | * | | * | | 7 | yes |
| Song ZW et al, 2013 | | * | | * | | * | |  | ** | | | |  |  | * | | * | | 7 | yes |
| Liu MM et al, 2014 | |  | |  | | * | |  | ** | | | |  |  | * | | * | | 5 | no |
| Xia YX, 2014 | |  | |  | | * | |  | * | | | |  |  | * | | * | | 4 | no |
| Lang HX et al, 2015 | | * | | * | | * | |  | * | | | |  |  | * | | * | | 6 | no |
| Zhang YH et al, 2015 | | * | | * | | * | |  | ** | | | |  |  | * | | * | | 7 | yes |
| Wu QL, 2016 | | * | | * | | * | |  |  | | | |  |  | * | | * | | 5 | no |
| Li XF, 2016 | | * | | * | | * | |  |  | | | |  |  | * | | * | | 5 | no |
| Qiao XW et al, 2016 | | * | | * | | * | |  | ** | | | |  |  | * | | * | | 7 | yes |
| Zheng L, 2017 | | * | | * | | * | |  | ** | | | |  |  | * | | * | | 7 | yes |
| Hu TL et al, 2017 | | * | | * | | * | |  |  | | | |  |  | * | | * | | 5 | no |
| Yu T et al, 2018 | | * | | * | | * | |  | ** | | | |  |  | * | | * | | 7 | yes |
| Ding XY et al, 2018 | | * | | * | | * | |  | ** | | | |  |  | * | | * | | 7 | yes |
| Yuan Y et al, 2018 | | * | | * | | * | |  | ** | | | |  |  | * | | * | | 7 | yes |
| Huang YM et al, 2018 | | * | | * | | * | |  | ** | | | |  |  | * | | * | | 7 | yes |
| Yang QH et al, 2019 | | * | | * | | * | |  | ** | | | |  |  | * | | * | | 7 | yes |
| Wang HT et al, 2020 | | * | | * | | * | |  | ** | | | |  |  | * | | * | | 7 | yes |
| Sun H et al, 2020 | |  | | * | | * | |  | * | | | |  |  | * | | * | | 5 | no |
| Chen ZT et al, 2020 | | * | | * | | * | |  | ** | | | |  |  | * | | * | | 7 | yes |
| Li SM et al, 2020 | | * | | * | | * | |  | ** | | | |  |  | * | | * | | 7 | yes |
| Zhao JJ, 2021 | | * | | * | | * | |  | ** | | | |  |  | * | | * | | 7 | yes |
| Sun LY et al, 2021 | | * | | * | | * | |  | ** | | | |  |  | * | | * | | 7 | yes |
| Li YJ, 2021 | | * | | * | | * | |  |  | | | |  |  | * | | * | | 5 | no |
| Hu Y et al, 2021 | | * | | * | | * | |  |  | | | |  | | * | | * | | 5 | no |
| Liang CC et al, 2021 | | * | | * | | * | |  |  | | | |  | | * | | * | | 5 | no |
| Long YX et al, 2022 | | * | | * | | * | | ** |  | | | |  | | * | | * | | 7 | yes |
| Pan YZ et al, 2022 | | * | | * | | * | | ** |  | | | |  | | * | | * | | 7 | yes |
| Yu T et al, 2022 | | * | | * | | * | | ** |  | | | |  | | * | | * | | 7 | yes |

* Note: A score of 7 or higher was considered good study.

**TABLE A4|** Sensitivity analysis of the prevalence of type Ⅰ violence

| Excluded study | Pooled prevalence (%, 95% CI) | I^2^ (%) |
| --- | --- | --- |
| Sha WW et al, 1995 | 23.33 (17.80-29.36) | 99.40 |
| Gan JL et al, 1997 | 23.42 (17.84-29.50) | 99.40 |
| Li YX, 2002 | 24.04 (18.37-30.20) | 99.40 |
| Lv JZ et al, 2002 | 23.22 (17.72-29.21) | 99.40 |
| Xiong YF et al, 2008 | 23.13 (17.68-29.07) | 99.40 |
| Ou MH et al, 2010 | 23.82 (18.13-30.02) | 99.40 |
| Ran MS et al, 2010 | 24.55 (18.95-30.60) | 99.40 |
| Gong CH et al, 2011 | 24.25 (18.57-30.41) | 99.40 |
| Wang FR et al, 2011 | 23.80 (18.11-29.99) | 99.40 |
| Xiong Y et al, 2011 | 24.38 (18.74-30.51) | 99.40 |
| Liang Y et al, 2012 | 22.70 (17.55-28.30) | 99.40 |
| Song ZW et al, 2013 | 24.13 (18.43-30.32) | 99.40 |
| Zhang JL et al, 2013 | 23.00 (17.61-28.88) | 99.40 |
| Chen SC et al, 2014 | 24.81 (19.35-30.71) | 99.40 |
| Zhang YH et al, 2015 | 23.39 (17.80-29.48) | 99.40 |
| Qiao XW et al, 2016 | 24.31 (18.65-30.46) | 99.40 |
| Zheng L, 2017 | 24.30 (18.64-30.45) | 99.30 |
| Ding XY et al, 2018 | 24.66 (19.11-30.66) | 99.30 |
| Huang YM et al, 2018 | 23.54 (17.97-29.60) | 99.30 |
| Yu T et al,2018 | 23.33 (17.80-29.36) | 99.40 |
| Yang QH et al, 2019 | 23.22 (17.72-29.21) | 99.40 |
| Wang HT et al, 2020 | 23.13 (17.68-29.07) | 98.90 |
| Li SM et al, 2020 | 23.82 (18.13-30.02) | 99.40 |
| Pan YZ et al, 2022 | 24.55 (18.95-30.60) | 99.00 |

*Note: a). Another method of sensitivity analysis is by removing each study individually in overall analysis.

b). There were 24 studies included in type Ⅰ violence.

**TABLE A5|** Sensitivity analysis of the prevalence of type Ⅱ violence

| Excluded study | Pooled prevalence (%, 95% CI) | I^2^ (%) |
| --- | --- | --- |
| Li YX, 2002 | 20.01 (4.22-43.22) | 97.30 |
| Xiong YF et al, 2008 | 22.05 (4.39-47.69) | 97.30 |
| Chen SC et al, 2014 | 17.30 (4.84-35.01) | 95.10 |
| Chen ZT et al, 2020 | 28.90 (11.61-50.09) | 97.40 |
| Long YX et al, 2022 | 28.31 (10.30-50.84) | 94.70 |

*Note: a). Another method of sensitivity analysis is by removing each study individually in overall analysis.

b). There were 5 studies included in type Ⅱ violence.

**Table A6|** Sensitivity analysis of the prevalence of type Ⅲ violence

| Excluded study | Pooled prevalence (%, 95% CI) | I^2^ (%) |
| --- | --- | --- |
| Cui Y et al, 1998 | 18.03 (8.45, 30.16) | 99.20 |
| Li YX, 2002 | 17.54 (8.09, 29.60) | 99.20 |
| Li HL et al, 2003 | 19.82 (10.91, 30.55) | 99.20 |
| Gao SW et al, 2006 | 16.88 (7.58, 28.85) | 99.20 |
| Ran MS et al, 2010 | 19.00 (9.59, 30.63) | 99.20 |
| Wei QL et al, 2011 | 15.52 (7.16, 26.29) | 99.20 |
| Yuan Y et al, 2018 | 15.58 (7.01, 26.67) | 98.80 |
| Sun LY et al, 2021 | 19.22 (9.90, 30.67) | 98.40 |
| Zhao JJ, 2021 | 15.16 (6.98, 25.70) | 99.10 |
| Long YX et al, 2022 | 17.21 (7.76, 29.33) | 99.20 |
| Yu T et al, 2022 | 15.48 (6.99, 26.45) | 98.90 |

*Note: a). Another method of sensitivity analysis is by removing each study individually in overall analysis.

b). There were 11 studies included in type Ⅲ violence.

**TABLE A7|** Sensitivity analysis of the prevalence of type Ⅳ violence

| Excluded study | Pooled prevalence (%) | I^2^ (%) |
| --- | --- | --- |
| Li HL et al, 2003 | 1.02 | - |
| Ran MS et al, 2010 | 0.26 | - |

*Note: a). Another method of sensitivity analysis is by removing each study individually in overall analysis.

b). There were 2 studies included in type Ⅳ violence.

**TABLE A8|** Pooled estimates of prevalence of type Ⅰ and type Ⅲ violence before and after trimming and filling

| Type of violence | Before | | |  | After | | |
| --- | --- | --- | --- | --- | --- | --- | --- |
|  | Pooled prevalence (%, 95% CI) | I^2^ (%) | Q-test P value |  | Pooled prevalence (%, 95% CI) | I^2^ (%) | Q-test P value |
| Ⅰ | 26.44 (20.62-32.68) | 99.40 | <0.01 |  | 11.94 (6.58-18.59) | 99.60 | <0.01 |
| Ⅲ | 17.19 (8.52-28.04) | 99.10 | <0.01 |  | 4.70 (0.01-15.28) | 99.30 | <0.01 |

**TABLE A9|** Spatial distribution of prevalence of violence (eligible studies)

| Type of violence | Study site | Number of data points | Pooled prevalence (%, 95% CI) |
| --- | --- | --- | --- |
| Ⅰ | Beijing | 2 | 25.57 (0.00-77.00) |
|  | Fujian | 1 | 24.37 |
|  | Gansu | 1 | 17.90 |
|  | Guangdong | 6 | 30.24 (8.60-58.08) |
|  | Guangxi | 4 | 28.15 (16.40-41.64) |
|  | Hainan | 1 | 39.80 |
|  | Henan | 5 | 34.77 (28.27-41.56) |
|  | Hubei | 1 | 18.74 |
|  | Jiangsu | 2 | 20.72 (1.12-54.54) |
|  | Inner Mongolia | 1 | 15.35 |
|  | Ningxia | 2 | 13.10 (6.99-20.72) |
|  | Shandong | 2 | 39.12 (32.08-46.40) |
|  | Sichuan | 3 | 19.80 (4.97-41.12) |
|  | Taiwan | 2 | 45.00 (25.56-65.24) |
|  | Yunnan | 1 | 11.31 |
|  | Zhejiang | 1 | 14.23 |
| Ⅱ | Beijing | 1 | 47.97 |
|  | Henan | 2 | 49.63 |
|  | Heilongjiang | 1 | 20.36 (15.83-25.29) |
|  | Hunan | 1 | 7.50 |
|  | Jiangsu | 2 | 35.16 (17.95-54.58) |
|  | Shandong | 2 | 24.76 (20.06-29.76) |
|  | Sichuan | 2 | 24.21 (17.47-31.66) |
|  | Taiwan | 2 | 24.07 (0.00-75.91) |
|  | Tibet | 1 | 61.02 |
| Ⅲ | Anhui | 1 | 36.78 |
|  | Beijing | 2 | 16.01 (8.49-25.29) |
|  | Fujian | 1 | 0.78 |
|  | Guangdong | 1 | 40.00 |
|  | Guangxi | 1 | 8.16 |
|  | Henan | 2 | 31.68 (2.90-72.21) |
|  | Hubei | 1 | 35.43 |
|  | Hunan | 1 | 17.25 |
|  | Liaoning | 1 | 41.29 |
|  | Shandong | 4 | 10.09 (8.89-11.35) |
|  | Sichuan | 1 | 3.89 |
| Ⅳ | Beijing | 1 | 14.13 |
|  | Fujian | 1 | 0.26 |
|  | Shandong | 1 | 1.60 |
|  | Sichuan | 1 | 1.02 |

**TABLE A10|** Subgroup analyses results (high-quality studies)

| Type of violence | | Ⅰ | | | |  | Ⅱ | | | |  | Ⅲ | | | |
| --- | --- | --- | --- | --- | --- | --- | --- | --- | --- | --- | --- | --- | --- | --- | --- |
| Classification | Subgroup | Number of data points | Pooled prevalence (%, 95%CI) | Q | I^2^ (%) |  | Number of data points | Pooled prevalence (%, 95%CI) | Q | I^2^ (%) |  | Number of data points | Pooled prevalence (%, 95%CI) | Q | I^2^ (%) |
| Sample source | Inpatient | 9 | 34.93 (26.54-43.82) | 110.12 | 92.70 |  | 3 | 38.35 (25.38-52.21) | 13.90 | 85.60 |  | 8 | 22.18 (10.94-35.93) | 467.56 | 98.50 |
|  | Non-inpatient | 15 | 18.40 (13.12-24.35) | 2838.71 | 99.50 |  | 2 | 6.95 (4.78-9.46) | 0.39 | 0.00 |  | 3 | 6.92 (1.13-16.95) | 98.65 | 98.00 |
| Publication year | ≤2012 | 11 | 24.42 (18.05-31.40) | 173.73 | 94.20 |  | 2 | 31.58 (22.32, 41.62) | 2.00 | 50.00 |  | 6 | 11.65 (3.16-24.19) | 118.40 | 95.80 |
|  | >2012 | 13 | 23.33 (14.97-32.90) | 3313.46 | 99.60 |  | 3 | 17.60 (0.46, 49.70) | 94.25 | 97.90 |  | 5 | 24.40 (10.12-42.41) | 995.85 | 99.60 |
| Age (year) | ≤40 | 9 | 29.83 (19.08-41.83) | 529.28 | 98.50 |  | 3 | 38.35 (25.38-52.21) | 13.90 | 85.60 |  | 6 | 25.83 (15.08-38.26) | 190.04 | 97.40 |
|  | >40 | 3 | 24.88 (8.60-45.88) | 39.32 | 94.90 |  | 2 | 6.95 (4.78-9.46) | 0.39 | 0.00 |  | 3 | 5.63 (0.13-17.67) | 92.97 | 97.80 |
| Proportion of male patients | ≤50% | 5 | 32.37 (15.48-51.97) | 391.66 | 99.00 |  | 4 | 22.05 (4.39-47.69) | 113.12 | 97.30 |  | 5 | 16.95 (7.55-29.06) | 205.31 | 98.10 |
|  | >50% | 7 | 26.06 (16.24-37.25) | 131.76 | 95.40 |  | 1 | 27.85 | 0.00 | - |  | 6 | 17.56 (4.32, 37.93) | 646.82 | 99.20 |
| Geographic distribution | Inland region | 17 | 24.44 (17.84-31.71) | 3450.96 | 99.50 |  | 3 | 22.36 (6.68-43.65)- | 55.13 | 96.40 |  | 8 | 20.88 (11.76-31.76) | 385.81 | 98.20 |
|  | Coastal areas | 7 | 22.36 (12.82-33.62) | 119.45 | 95.00 |  | 2 | 24.07 (0.00-75.91) | 54.78 | 98.20 |  | 2 | 13.72 (0.00-67.98) | 51.42 | 98.10 |
| Economic circle | Economic circles | 8 | 21.36 (11.82-32.79) | 2360.20 | 99.70 |  | 0 | - | - | - |  | 2 | 17.10 (7.14-30.17) | 551.95 | 98.7 |
|  | Non-economic circles | 14 | 22.99 (16.92-29.68) | 669.06 | 98.10 |  | 3 | 22.36 (6.68-43.65) | 55.13 | 96.4 |  | 8 | 29.01 (12.24-49.30) | 5.22 | 80.8 |
| Combination of geographic distribution and economic circle | Inland region | 17 | 24.44 (17.84-31.71) | 3450.96 | 99.50 |  | 3 | 22.36 (6.68-43.65) | 55.13 | 96.4 |  | 8 | 20.88 (11.76-31.76) | 385.81 | 98.2 |
|  | Coastal economic zone | 3 | 17.99 (12.00-24.89) | 36.76 | 94.60 |  | 0 | - | - | - |  | 1 | 40.00 | 0.00 | - |
|  | Coastal non-economic zone | 2 | 12.86 (10.38-15.55) | 0.52 | 0.0 |  | 0 | - | - | - |  | 1 | 0.78 | 0.00 | - |

*Note: This symbol “-” indicates that data is note available.

† We didn’t perform subgroup analyses on some indicators (i.e., course, proportion of married patients and proportion of patient with family history of mental disorders) of which included articles was fewer than 4.

**TABLE A11|** Subgroup analyses results (eligible studies)

| Type of violence | | Ⅰ | | | |  | Ⅱ | | | |  | Ⅲ | | | |
| --- | --- | --- | --- | --- | --- | --- | --- | --- | --- | --- | --- | --- | --- | --- | --- |
| Classification | Subgroup | Number of data points | Pooled prevalence (%, 95%CI) | Q | I^2^ (%) |  | Number of data points | Pooled prevalence (%, 95%CI) | Q | I^2^ (%) |  | Number of data points | Pooled prevalence (%, 95%CI) | Q | I^2^ (%) |
| Sample source | Inpatient | 17 | 35.67 (25.98-45.98) | 1779.54 | 99.10 |  | 11 | 34.32 (26.32-42.78) | 103.61 | 90.30 |  | 14 | 18.72 (10.89-28.04) | 586.05 | 97.80 |
|  | Non-inpatient | 18 | 18.83 (13.90-24.31) | 2884.37 | 99.40 |  | 3 | 20.13 (0.01-59.68) | 82.18 | 97.60 |  | 3 | 6.92 (1.13-16.95) | 98.65 | 98.00 |
| Publication year | ≤2012 | 16 | 25.97 (20.29-32.08) | 321.56 | 95.30 |  | 6 | 29.17 (20.91-38.17) | 41.70 | 88.00 |  | 10 | 10.85 (5.76-17.25) | 134.82 | 93.30 |
|  | >2012 | 19 | 26.81 (17.38-37.42) | 6899.47 | 99.70 |  | 8 | 32.72 (17.14-50.51) | 237.36 | 97.10 |  | 7 | 24.54 (11.05-41.22) | 1101.58 | 99.50 |
| Age (year) | ≤40 | 13 | 28.71 (20.45-37.76) | 625.01 | 98.10 |  | 7 | 36.32 (27.87-45.21) | 42.25 | 85.80 |  | 9 | 19.15 (10.81-29.15) | 229.30 | 96.50 |
|  | >40 | 3 | 24.88 (8.60-45.88) | 39.32 | 94.9 |  | 2 | 6.95 (4.78-9.46) | 0.39 | 0.00 |  | 4 | 6.63 (1.27-15.57) | 98.32 | 96.90 |
| Proportion of male patients | ≤50% | 5 | 32.37 (15.48-51.97) | 391.66 | 99.00 |  | 4 | 22.05 (4.39-47.69) | 113.12 | 97.30 |  | 5 | 16.95 (7.55-29.06) | 205.31 | 98.10 |
|  | >50% | 14 | 26.90 (20.35-34.01) | 275.69 | 95.30 |  | 9 | 32.29 (23.52-41.72) | 83.43 | 90.40 |  | 11 | 16.50 (7.31-28.40) | 784.36 | 98.70 |
| Geographic distribution | Inland region | 26 | 24.41 (19.32-29.88) | 3744.26 | 99.30 |  | 12 | 32.27 (22.84-42.48) | 228.77 | 95.20 |  | 13 | 18.71 (11.31-27.44) | 501.09 | 97.60 |
|  | Coastal areas | 9 | 32.58 (15.60-52.30) | 2569.15 | 99.70 |  | 2 | 24.07 (0.00-75.91) | 54.78 | 98.20 |  | 3 | 12.27 (0.00-41.14) | 68.44 | 97.10 |
| Economic circle | Economic circles | 8 | 21.36 (11.82-32.79) | 2360.20 | 99.70 |  | 3 | 39.59 (26.07-53.95) | 14.96 | 86.60 |  | 3 | 22.46 (9.19-39.29) | 14.20 | 85.90 |
|  | Non-economic circles | 25 | 26.94 (19.90-34.61) | 3561.40 | 99.30 |  | 9 | 29.99 (18.72-42.62) | 176.68 | 95.50 |  | 13 | 16.29 (8.60-25.80) | 646.25 | 98.10 |
| Combination of geographic distribution and economic circle | Inland region | 26 | 24.41 (19.32-29.88) | 3744.26 | 99.30 |  | 12 | 32.27 (22.84-42.48) | 228.77 | 95.20 |  | 13 | 18.71 (11.31-27.44) | 501.09 | 97.60 |
|  | Coastal economic zone | 3 | 17.99 (12.00-24.89) | 36.76 | 94.60 |  | 0 | - | - | - |  | 1 | 40.00 | 0.00 | - |
|  | Coastal non-economic zone | 4 | 39.55 (6.18-80.04) | 1458.27 | 99.80 |  | 0 | - | - | - |  | 2 | 4.20 (0.00-18.05) | 28.51 | 96.50 |

*Note: This symbol “-” indicates that data is note available.

† We didn’t perform subgroup analyses on some indicators (i.e., course, proportion of married patients and proportion of patient with family history of mental disorders) of which included articles was fewer than 4.

**TABLE A12|** Variables Assignment Table

| Variables | Meaning | Assignment | |
| --- | --- | --- | --- |
| source | Sample source | 1: inpatient | 0: non-inpatient |
| publication | Publication year | 1: ≤2012 | 0: >2012 |
| geometric | Geographic distribution | 1: inland area | 0: coastal area |
| economic | Economic circle | 1: economic circle | 0: non-economic circle |
| age | Age (years) | 1: ≤40 | 0: >40 |
| male | Proportion of male patients | 1: ≤0.5 | 0: >0.5 |
| course | Disease course (years) | 1: ≤10 | 0: >10 |
| married | Proportion of married patients | 1: ≤0.5 | 0: >0.5 |
| family | Proportion of patient with family history of mental disorders | 1: ≤0.5 | 0: >0.5 |
| GDP | GDP per capita: per capita gross domestic product (￥10000) | quantitative variables | |
| unemployment | Unemployment rate: total unemployment / total population | quantitative variables | |
| burden | Total burden coefficient: total population aged <15 and ≥65 / total population aged 15-65 | quantitative variables | |
| illiteracy | Illiteracy ratio: illiterate population aged ≥15/ total population aged ≥15 | quantitative variables | |
| bed | Beds per 1,000 population in health institutions | quantitative variables | |

**TABLE A13|** The results of univariate meta-regression analyses (high-quality studies)

| Type of violence | Ⅰ | | | |  | Ⅱ | | | |  | Ⅲ | | | |
| --- | --- | --- | --- | --- | --- | --- | --- | --- | --- | --- | --- | --- | --- | --- |
| Moderators | Number of data points | Coefficient (β) | Coefficient P value | R^2^ (%) |  | Number of data points | Coefficient (β) | Coefficient P value | R^2^ (%) |  | Number of data points | Coefficient (β) | Coefficient P value | R^2^ (%) |
| Sample source (inpatient/non-inpatient) | 24 | 0.19 | <0.01 | 28.21 |  | 5 | 0.41 | <0.01 | 85.99 |  | 11 | 0.22 | 0.10 | 15.04 |
| Publication year (≤2012/>2012) | 24 | 0.02 | 0.83 | 0.00 |  | 5 | 0.17 | 0.45 | 0.00 |  | 11 | -0.16 | 0.20 | 7.01 |
| Geographic distribution (inland area/coastal area) | 24 | 0.02 | 0.76 | 0.00 |  | 5 | -0.02 | 0.94 | 0.00 |  | 10 | 0.10 | 0.56 | 0.00 |
| Economic circle (economic circle/non-economic circle) | 22 | -0.02 | 0.77 | 0.00 |  | 3 | - | - | - |  | 10 | -0.15 | 0.39 | 0.00 |
| Age (≤40/>40) | 12 | 0.05 | 0.69 | 0.00 |  | 5 | 0.41 | <0.01 | 85.99 |  | 9 | 0.29 | 0.01 | 41.35 |
| Proportion of male patients (≤0.5/>0.5) | 12 | 0.07 | 0.53 | 0.00 |  | 5 | -0.07 | 0.83 | 0.00 |  | 11 | -0.01 | 0.96 | 0.00 |
| Disease course (≤10/>10) | 4 | 0.27 | 0.12 | 34.26 |  | 1 | - | - | - |  | 5 | 0.37 | 0.02 | 55.23 |
| Proportion of married patients (≤0.5/>0.5) | 4 | 0.35 | 0.22 | 14.78 |  | 2 | - | - | - |  | 4 | 0.09 | 0.74 | 0.00 |
| Proportion of patient with family history of mental disorders (≤0.5/>0.5) | 5 | 0.09 | 0.30 | 3.66 |  | 2 | - | - | - |  | - | - | - | - |
| GDP per capita ($10000) | 23 | -0.01 | 0.50 | 0.00 |  | 4 | -0.01 | 0.50 | 0.00 |  | 7 | 0.07 | <0.01 | 55.80 |
| Unemployment rate | 23 | 2.70 | 0.49 | 0.00 |  | 4 | -4.44 | 0.85 | 0.00 |  | 7 | -22.83 | <0.01 | 73.76 |
| Total burden coefficient | 20 | 1.00 | 0.02 | 21.82 |  | 3 | 5.29 | 0.30 | 2.44 |  | 7 | 0.00 | 0.97 | 0.00 |
| Illiteracy rate | 20 | 0.62 | 0.36 | 0.00 |  | 2 | - | - | - |  | 7 | -1.65 | 0.18 | 11.51 |
| Beds per 1,000 population in health institutions | 17 | -0.03 | 0.33 | 0.00 |  | 2 | - | - | - |  | 6 | 0.07 | 0.14 | 21.07 |

*Note: This symbol “-” indicates that the covariate cannot be entered the regression model.

**TABLE A14|** The results of univariate meta-regression analyses (eligible studies)

| Type of violence | Ⅰ | | | |  | Ⅱ | | | |  | Ⅲ | | | |
| --- | --- | --- | --- | --- | --- | --- | --- | --- | --- | --- | --- | --- | --- | --- |
| Moderators | Number of data points | Coefficient (β) | Coefficient P value | R^2^ (%) |  | Number of data points | Coefficient (β) | Coefficient P value | R^2^ (%) |  | Number of data points | Coefficient (β) | Coefficient P value | R^2^ (%) |
| Sample source (inpatient/non-inpatient) | 35 | 0.19 | <0.01 | 20.55 |  | 14 | 0.17 | 0.20 | 5.88 |  | 17 | 0.18 | 0.15 | 6.50 |
| Publication year (≤2012/>2012) | 35 | ＜-0.01 | 0.91 | 0.00 |  | 14 | -0.04 | 0.76 | 0.00 |  | 17 | -0.17 | 0.07 | 14.11 |
| Geographic distribution (inland area/coastal area) | 35 | -0.09 | 0.26 | 0.79 |  | 14 | 0.09 | 0.58 | 0.00 |  | 16 | 0.09 | 0.49 | 0.00 |
| Economic circle (economic circle/non-economic circle) | 33 | 0.07 | 0.43 | 0.00 |  | 12 | - | - | - |  | 16 | 0.08 | 0.53 | 0.00 |
| Age (≤40/>40) | 16 | 0.04 | 0.72 | 0.00 |  | 9 | 0.386 | ＜0.01 | 73.21 |  | 13 | 0.19 | 0.06 | 19.07 |
| Proportion of male patients (≤0.5/>0.5) | 19 | 0.06 | 0.49 | 0.00 |  | 13 | -0.12 | 0.31 | 0.35 |  | 16 | 0.01 | 0.96 | 0.00 |
| Disease course (≤10/>10) | 6 | 0.10 | 0.49 | 0.00 |  | 2 | - | - | - |  | 7 | 0.26 | 0.11 | 21.86 |
| Proportion of married patients (≤0.5/>0.5) | 7 | 0.17 | 0.35 | 0.00 |  | 4 | -0.17 | 0.58 | 0.00 |  | 6 | -0.11 | 0.62 | 0.00 |
| Proportion of patient with family history of mental disorders (≤0.5/>0.5) | 7 | 0.01 | 0.91 | 0.00 |  | 4 | - | - | - |  | 3 | - | - | - |
| GDP per capita ($10000) | 32 | ＜0.01 | 0.50 | 0.00 |  | 12 | ＜-0.01 | 0.73 | 0.00 |  | 13 | 0.04 | 0.04 | 22.31 |
| Unemployment rate | 32 | 1.08 | 0.75 | 0.00 |  | 12 | -6.81 | 0.35 | 0.00 |  | 13 | -14.36 | 0.03 | 25.40 |
| Total burden coefficient | 28 | 1.04 | 0.01 | 17.68 |  | 10 | -1.17 | 0.13 | 15.79 |  | 12 | ＜0.01 | 0.87 | 0.00 |
| Illiteracy rate | 28 | 0.31 | 0.64 | 0.00 |  | 9 | -0.20 | 0.32 | 0.00 |  | 12 | -1.21 | 0.16 | 7.58 |
| Beds per 1,000 population in health institutions | 24 | -0.03 | 0.25 | 1.44 |  | 6 | 0.02 | 0.56 | 0.00 |  | 10 | 0.04 | 0.16 | 12.50 |

*Note: This symbol “-” indicates that the covariate cannot be entered the regression model.

**TABLE A15|** The results of multivariate meta-regression analysis (eligible studies)

| **Type of violence** | **Moderators** | **Number of data points** | **Coefficient (β)** | **Coefficient P value** | **R^2^ (%)** |
| --- | --- | --- | --- | --- | --- |
| Ⅰ | Sample source (inpatient/non-inpatient) | 28 | 0.13 | <0.01 | 37.91 |
|  | Total burden coefficient |  | 0.69 | 0.07 |  |
| Ⅱ | Sample source (inpatient/non-inpatient) | 13 | 0.47 | <0.01 | 53.01 |
|  | Proportion of male patients (≤0.5/>0.5) |  | 0.13 | 0.25 |  |
| Ⅲ | Age (≤40/>40) | 10 | 0.16 | 0.07 | 25.62 |
|  | GDP per capita ($10000) |  | 0.01 | 0.42 |  |

**
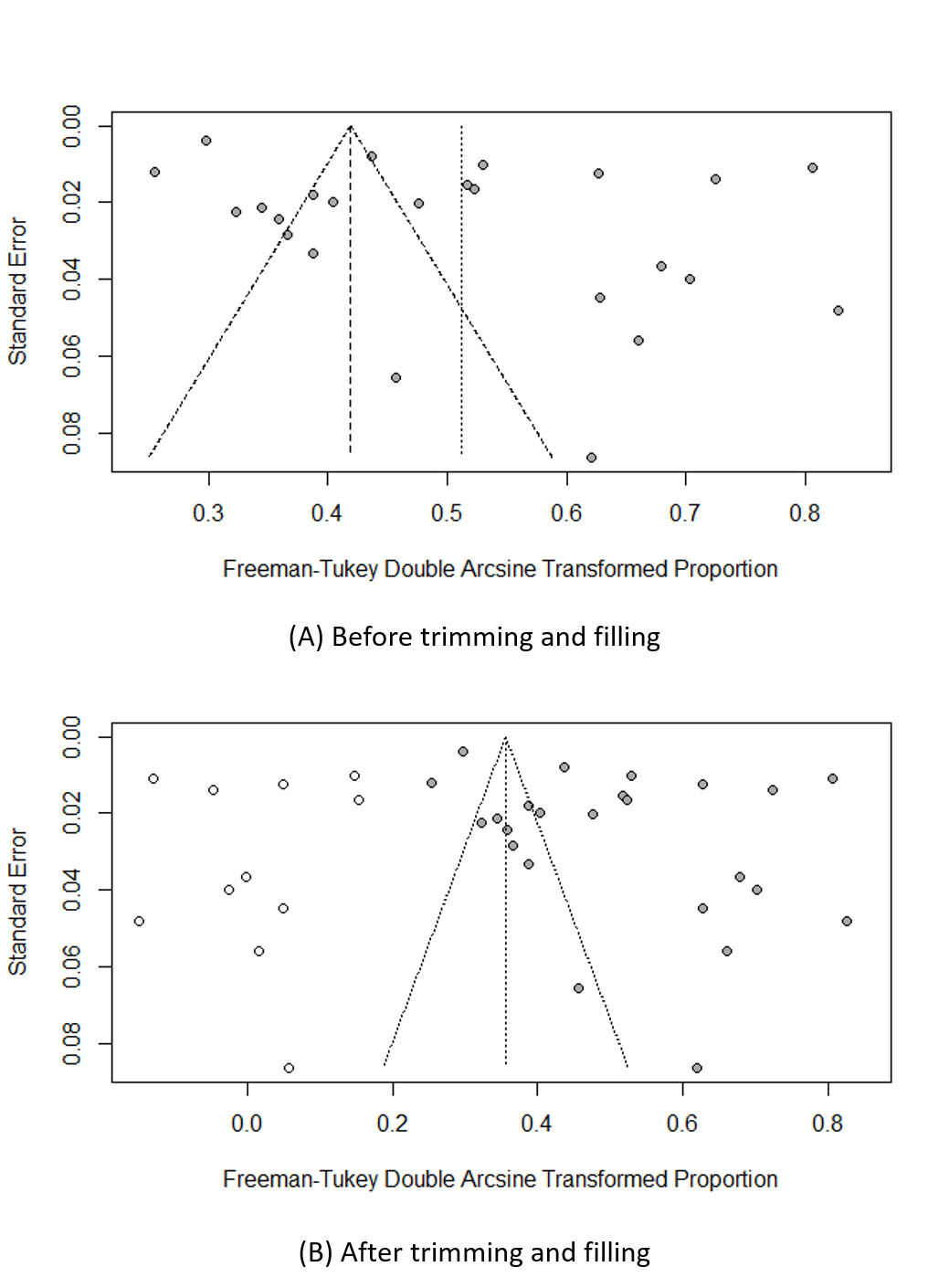
**

**FIGURE A1|** Funnel plot of prevalence of type Ⅰ violence


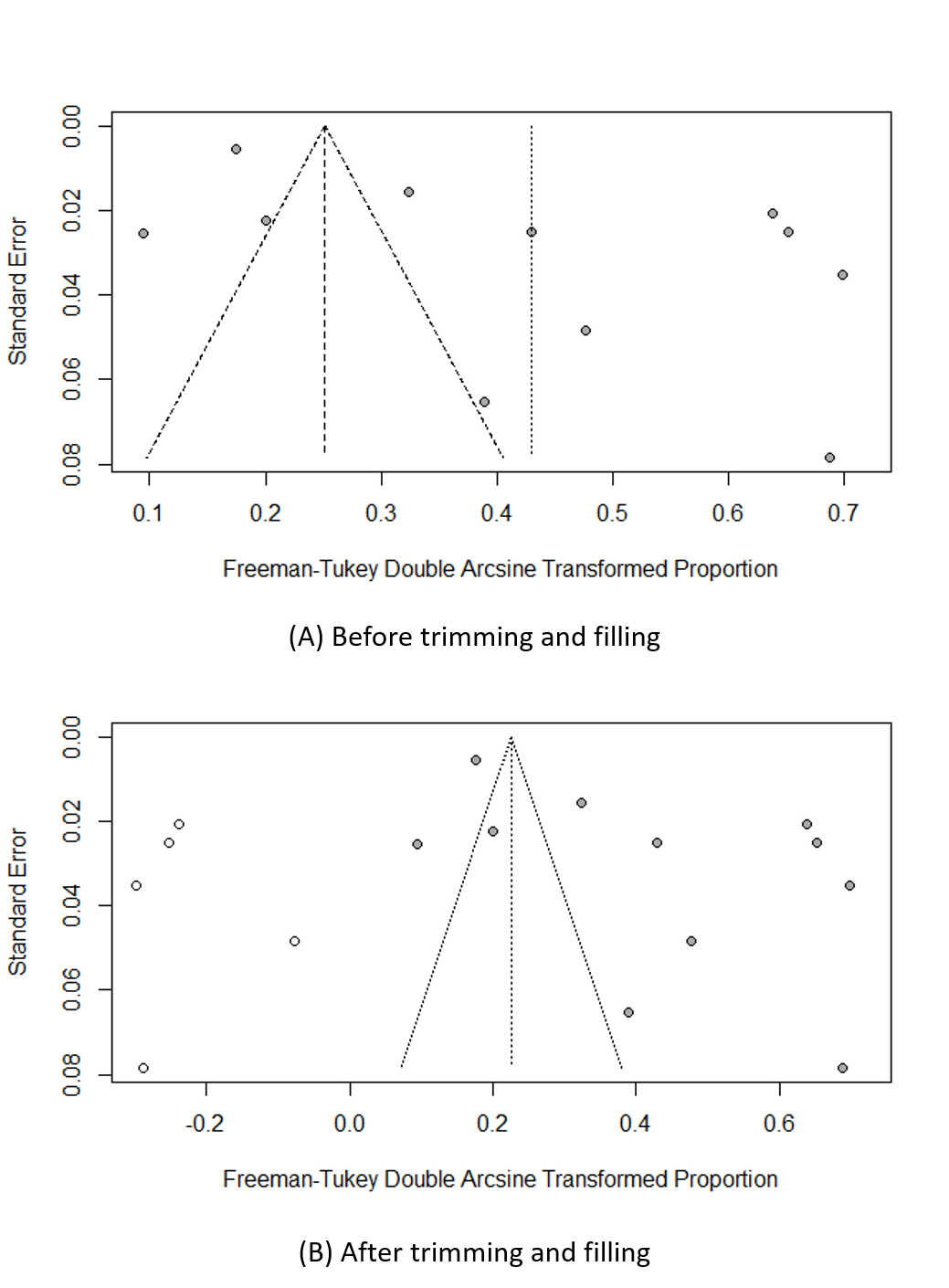


**FIGURE A2|** Funnel plot of prevalence of type Ⅲ violence
